# Increase of Salt Tolerance in Carbon-Starved Cells of *Rhodopseudomonas palustris* Depending on Photosynthesis or Respiration

**DOI:** 10.3390/microorganisms6010004

**Published:** 2018-01-06

**Authors:** Sawa Wasai, Nanako Kanno, Katsumi Matsuura, Shin Haruta

**Affiliations:** Department of Biological Sciences, Tokyo Metropolitan University, 1-1 Minami-Osawa, Hachioji, Tokyo 192-0397, Japan; arai.sawa@ige.tohoku.ac.jp (S.W.); n_kanno@tmu.ac.jp (N.K.); matsuura-katsumi@tmu.ac.jp (K.M.)

**Keywords:** carbon starvation, salt stress tolerance, cellular energy, purple photosynthetic bacteria

## Abstract

Bacteria in natural environments are frequently exposed to nutrient starvation and survive against environmental stresses under non-growing conditions. In order to determine the energetic influence on survivability during starvation, changes in salt tolerance were investigated using the purple photosynthetic bacterium *Rhodopseudomonas palustris* after carbon starvation under photosynthetic conditions in comparison with anaerobic and aerobic dark conditions. Tolerance to a treatment with high concentration of salt (2.5 M NaCl for 1 h) was largely increased after starvation under anaerobically light and aerobically dark conditions. The starved cells under the conditions of photosynthesis or aerobic respiration contained high levels of cellular ATP, but starvation under the anaerobic dark conditions resulted in a decrease of cellular ATP contents. To observe the large increase of the salt tolerance, incubation of starved cells for more than 18 h under illumination was needed. These results suggest that the ATP-dependent rearrangement of cells induced salt tolerance.

## 1. Introduction

Bacteria are often exposed to nutrient-depleted conditions in natural environments [1]. Bacterial functions and strategies for survival and distribution in nature are strongly related to nutrient-depleted conditions. Bacteria likely change their cellular metabolisms to adapt to nutrient-depleted conditions [2]. Bacterial behaviors under carbon-starved non-growing conditions have been studied extensively in *Escherichia coli* and other bacteria in human microbiota [3,4,5,6,7,8]. Recently, much attention has been paid to bacteria in natural environments as well [9]. As a pronounced cellular response to nutrient starvation, a cell size reduction has been observed [10,11,12,13,14,15,16,17]. Recently, Wu et al. reported the reduction of ATP levels in starved cells of mycobacteria [17]. Other studies reported that bacteria under starved conditions had increased tolerance against environmental stresses, e.g., salt, hyperosmotic pressure, desiccation, heat, and ultraviolet (UV) radiation [3,16,18]. Transcriptional factors responding to the starvation have also been extensively examined [19,20,21,22]. However, it is still unclear how cellular energy contributes to these starvation responses.

Purple photosynthetic bacteria are able to convert light energy to ATP by anoxygenic cyclic photophosphorylation. It has been reported that illumination prolongs the viability of purple photosynthetic bacteria [23,24,25]. In addition, Kanno et al. previously examined ATP contents in purple photosynthetic bacteria, *Rhodopseudomonas palustris* and *Rhodospirillum rubrum*, under carbon-starved conditions and reported that illumination largely affected the maintenance of cellular ATP contents under carbon-starved conditions [24]. These results suggest that cellular ATP is utilized to increase the survivability of the purple photosynthetic bacteria. In this study, we prepared carbon-starved cells of the purple photosynthetic bacterium *R. palustris*, which is widely distributed and metabolically versatile [26], and we investigated changes in salt tolerance after short-term starvation under photosynthetic conditions, as compared with anaerobic and aerobic dark conditions.

## 2. Materials and Methods

### 2.1. Bacterial Growth and Preparation of Carbon-Starved Cells

*R. palustris* ATCC BAA-98 (= CGA009) was obtained from the American Type Culture Collection. This bacterial strain was anaerobically pre-cultured under illumination at 30 °C in a carbon-rich medium [24]. To obtain carbon-starved cells, 1 mL of culture at the early stationary phase of growth was inoculated into 20 mL of a carbon-limited medium in 32-mL glass test tubes. The glass test tubes were sealed with butyl rubber stoppers and screw caps after replacing the gas phase with N_2_ gas. The carbon-limited medium contained 0.5 g/L of disodium succinate hexahydrate as a sole carbon source as described previously [24]. The anaerobic culture was illuminated (tungsten lamp with a 750-nm longpass filter) at 30 °C and the growth stopped at an optical density of approximately 0.3 (Figure S1), although the optical density reached 1.0 in the carbon-rich medium, as reported previously [24]. We defined the exponentially growing cells approximately 2 h before growth termination as “growing cells”. The cells that showed no further growth were defined as “starvation-initiated cells”. The starvation-initiated cells were incubated for 24 h either in lighted or darkened conditions. In addition, the starvation-initiated cells were transferred into T-shaped test tubes equipped with a silicon plug (Silicosen, Shin-etsu Polymer, Tokyo, Japan) and were aerobically incubated with shaking at 120 rpm in the dark.

### 2.2. Cellular ATP Contents

Cellular ATP contents were determined as follows: bacterial cells in 100 µL of culture were collected by centrifugation after suspension in 900 µL of pre-chilled 50 mM HEPES buffer (pH 7.0). The cells were suspended in 400 µL of 0.1 M HNO_3_ and incubated on ice without illumination for 10 min to extract cellular ATP. Then, 500 µL of 0.185 M Tris were added and the samples were stored at 4 °C until measurement. The ATP concentration in the sample was measured using ATP Bioluminescence Assay Kit CLS II (Roche Diagnostics GmbH, Mannheim, Germany) and a microplate reader (infinite M200 PRO, Tecan, Seestrasse, Switzerland). 

### 2.3. Evaluation of Salt Stress Tolerance

Salt stress tolerance was evaluated as follows: one mL of culture solution was added into 4 mL of the carbon-free medium supplemented with NaCl (final concentration, 2.5 M) in a 32-mL test tube that was sealed with a butyl rubber stopper and a screw cap after replacing the gas phase with N_2_ gas. The tubes were incubated for 1 h at 30 °C in the dark. Viability was determined by colony counting using PYS (5.0 g polypeptone, 1.0 g yeast extract, 5.0 g sodium succinate, 1.0 g casamino acids, 1.0 g (NH_4_)_2_SO_4_, 0.38 g KH_2_PO_4_, 0.39 g K_2_HPO_4_, and 1 mL basal salt solution [27]) agar plates. A series of 5-fold dilutions were prepared using the carbon-free medium. Three agar plates were used per one of the serial dilutions. The agar plates were cultivated for approximately 7 days at 30 °C in the dark, and the number of colonies was counted. The percent survival was calculated as a percentage of colony forming units (CFUs) without exposure to NaCl stress. 

### 2.4. Microscopic Analysis of Cells

Phase-contrast images of cells were captured with optical microscope (Axio Imager 2, Carl Zeiss, Oberkochen, Germany) equipped with a camera DP73 (Olympus, Tokyo, Japan). The length of the long axis of rod-shaped cells was measured with cellSens imaging software (cellSens standard, Olympus). The length of 150 cells were measured for each sample with three replications.

## 3. Results

### 3.1. Cellular ATP Contents

The exponentially growing cells and the growth-terminated cells due to carbon depletion (i.e., the starvation-initiated cells) were prepared as described above. The starvation-initiated cells were incubated in the light and dark to prepare 1-d starved cells in the light and 1-d starved cells in the dark, respectively. Their colony forming unit (CFU) values were not significantly changed by the 1-d starvation (*p* > 0.05 by the Student’s *t*-test, data not shown), as previously shown. As summarized in Table 1, cellular ATP contents slightly increased from (4.12 ± 0.52) × 10^−16^ mol CFU^-1^ in the growing cells to (6.45 ± 0.37) × 10^−16^ mol CFU^−1^ in the growth-terminated cells. This was likely due to the reduction of ATP consumption caused by growth termination. ATP content significantly increased to (12.9 ± 0.87) × 10^−16^ mol CFU^−1^ and decreased to (4.06 ± 0.45) × 10^−16^ mol CFU^−1^ after 1-d anaerobic starvation in the light and the dark, respectively. As suggested previously [23,28], ATP is probably consumed to maintain viability even under carbon starvation conditions, but in the case of this bacterium ATP can be photosynthetically produced in the light. When aerobic dark conditions were applied after growth termination, the 1-d aerobic starved cells showed high ATP contents, (7.49 ± 0.11) × 10^−16^ mol CFU^−1^, in contrast to the anaerobic starved cells in the dark. This probably indicates that oxygen supply allows the cells to maintain cellular ATP levels through oxidative phosphorylation using a part of cellular organic compounds as the source of electron donors.

### 3.2. Salt Stress Tolerance

**Figure 1:**
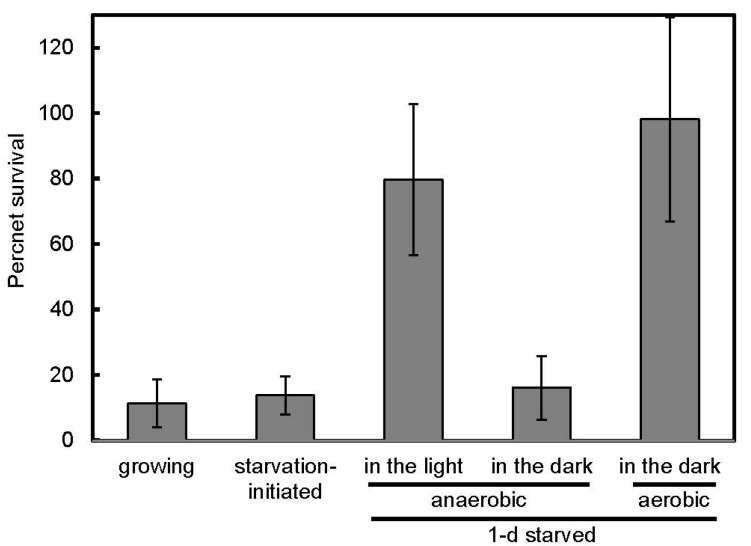
Survivability after exposure to salt stress. CFU values after incubation in 2.5 M NaCl for 1 h in the dark were determined for exponentially growing cells (growing), starvation-initiated cells (starvation-initiated), 1-d anaerobic starved cells in the light, 1-d anaerobic starved cells in the dark, and 1-d aerobic starved cells in the dark. The percent survival was calculated as a percentage of CFUs after incubation without NaCl. Error bars indicate standard deviation of three or four biological replicates.

Salt stress tolerance was evaluated after exposure to 2.5 M NaCl for 1 h (Figure 1). Upon the application of stress to the growing cells, the percent survival was 11.3%. The sensitivity to the salt stress was nearly unchanged in the starvation-initiated cells: only less than 15% of starvation-initiated cells were tolerant. We reported previously that the starvation-initiated cells of *R. palustris* CGA009 were resistant to 10-min incubation in 2.5 M NaCl [24]. The present results indicate that the prolonged exposure to 2.5 M NaCl for 1 h decreased viability of the starved cells. 

**Figure 2:**
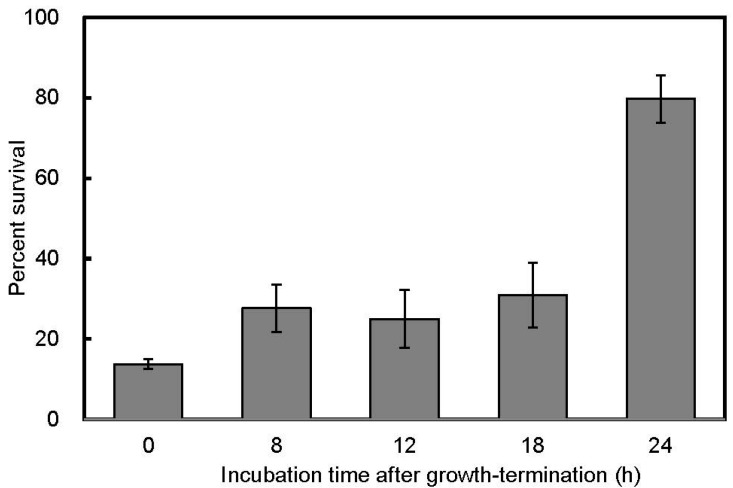
Changes in salt stress tolerance of starved cells in the light. As shown in Figure 1, the percent survival after exposure to 2.5 M NaCl stress were determined for starvation-initiated cells (i.e., 0 h starved cells) and anaerobic starved cells incubated for 8 h, 12 h, 18 h, and 24 h in the light. Error bars indicate standard deviation of three biological replicates.

Although salt stress tolerance in 1-d anaerobic starved cells in the dark did not increase (the survival ratio was 16.1%), a marked increase in the salt stress tolerance was observed in 1-d anaerobic starved cells in the light and 1-d aerobic starved cells in the dark: their percent survival were 79.7% and 98.1%, respectively (Figure 1). Changes in the salt stress tolerance of starved cells in the light were determined during a 24-h incubation after growth termination (Figure 2). The percent survival did not largely change until 18 h of incubation, and significantly increased at 24 h.

### 3.3. Cell Size

**Figure 3:**
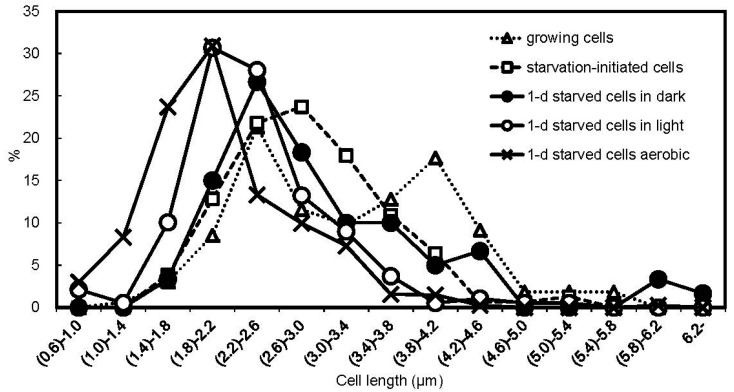
Frequency distribution of cell length. The length of the long axis of rod-shaped cells was measured for exponentially growing cells, starvation-initiated cells, 1-d anaerobic starved cells in the light, 1-d anaerobic starved cells in the dark, and 1-d aerobic starved cells in the dark. The frequency of the cell lengths is represented as a percentage of the total cells measured. The lengths of 150 cells were measured for each sample with three replications. The horizontal axis indicates cell length (μm), and the percentages of cells with lengths in the range in total cells measured are plotted, e.g., (0.6)–1.0 indicates a range from longer than 0.6 μm to 1.0 μm.

The cell length distribution was measured in the exponentially growing cells, starvation-initiated cells, 1-d anaerobic starved cells in the light, 1-d anaerobic starved cells in the dark, and 1-d aerobic starved cells in the dark with phase-contrast microscopy (Figure 3). In the growing culture, there were two peaks in the distribution of cell length: the shorter cells seem to be cells just after cell division, and the longer cells could be elongated cells before cell division. In the starvation-initiated cultures, more than 60% of the cells had cell lengths in the range from 2.2 to 3.4 µm. Cell length after 1-d anaerobic starvation in the dark was not markedly changed as compared to starvation initiation. However, 1-d anaerobic starvation in the light shortened the cell length, i.e., most cells in the 1-d anaerobic starved culture in the light had lengths of 1.4–2.6 µm (72%). Similarly, shorter cells appeared after 1-d aerobic starvation in the dark.

## 4. Discussion

In this study, the carbon-starved cells of *R. palustris* CGA009 were incubated for 24 h (1) anaerobically in the light (photosynthetic conditions); (2) aerobically in the dark (respiratory conditions); or (3) anaerobically in the dark in the absence of a terminal electron acceptor. We found that cellular ATP levels in this bacterium were decreased by 1-d starvation in the dark, but the ATP levels in the starved cells in the light and the aerobic starved cells were increased (Table 1). The latter two types of starved cells showed high tolerance to the salt stress (Figure 1). In the salt-tolerant cells, the cell length was markedly shortened (Figure 3), supporting the rearrangement of cellular components during the starved incubation of cells with the energy supply. It has been suggested that cell size reduction is an adaptation response to unfavorable conditions [29] and that also seems to be the case in this study. The large increase in the salt tolerance under the conditions of energy supply may be related to changes in cells in preparation for possibly forthcoming large stresses in natural environments.

Cellular ATP can be used to drive ion efflux pumps to maintain cellular osmolality [30,31]. However, it is unlikely that ATP-powered transporters largely contributed to the resistance to the salt stress in the starved cells of *R. palustris*, since more than 75% of total cellular ATP in 1-d starved cells disappeared within 10 min when the cells were exposed to NaCl, and the ATP contents after the decrease were less than those in the sensitive cells (data not shown). Oren reported that cellular ATP leaks from cells under hyperosmotic pressure [30]. During the starvation period, it is likely that ATP was used to make cells tolerant against the stress and the rearrangement needed sufficient time as indicated in Figure 2. It has been suggested that tolerance to high osmolality is increased by reconstruction of cytoplasmic membrane [32,33,34] or cell wall [35]. These changes in the membrane or cell wall could be accompanied by the reduction of cell size, which was observed in the tolerant cells (Figure 3). High accumulation of a compatible solute such as trehalose and glycine betaine in cytoplasm does not seem to occur under the conditions where available carbon sources are limited [36,37]. 

Our results strongly indicate that cellular energy influences starvation responses. ATP production through photosynthesis and aerobic respiration probably supports the adaptive changes. Carbon-starvation-induced changes have been reported for several species of non-photosynthetic aerobic bacteria [3], and most of those studies applied aerobic conditions to starved cells [11,16,38,39,40]. Similar effects of anaerobic illumination and oxygen supply on the salt tolerance in this study indicate that the energy supply is a major factor in rearranging the non-growing cells for stress tolerance. Aerobic respiration may also be helpful for the starvation responses in various bacteria, as shown in this study. These could provide an important aspect with respect to the survivability of bacteria in natural environments. Bacteria under non-growing states appear to actively respond to environments, and manage the synthesis and utilization of their cellular energy to not only to maintain their viability under the given conditions but also to prepare the cells for further environmental changes.

## Figures and Tables

**Table 1:** Cellular ATP contents.

	Growing Cells	Starvation-Initiated Cells	1-d Anaerobic Starved Cells in the Light	1-d Anaerobic Starved Cells in the Dark	1-d Aerobic Starved Cells in the Dark
× 10^−16^ mol CFU^−1^	4.12 ± 0.52	6.45 ± 0.37	12.9 ± 0.87	4.06 ± 0.45	7.49 ± 0.11

## Supplementary Materials

The following are available online at http://www.mdpi.com/2076-2607/6/1/4/s1, Figure S1: Phototrophic growth of *R. palustris* CGA009 in the carbon-limited medium.

Supplementary File 1
