# Supplementary material for: Increase of Salt Tolerance in Carbon-Starved Cells of Rhodopseudomonas palustris Depending on Photosynthesis or Respiration
Source: Microorganisms. 2018 Jan 6;6(1):4. doi: 10.3390/microorganisms6010004 (PMC5874618; doi:10.3390/microorganisms6010004)

Supplemental Material

Figure S1. Phototrophic growth of *R. palustris* CGA009 in the carbon-limited medium

We used the cells approximately 2 hours before the growth-termination as “growing cells” (indicated by upward arrow). The cells, indicated by downward arrow, were defined as “starvation-initiated cells” after no increase of the OD was confirmed.

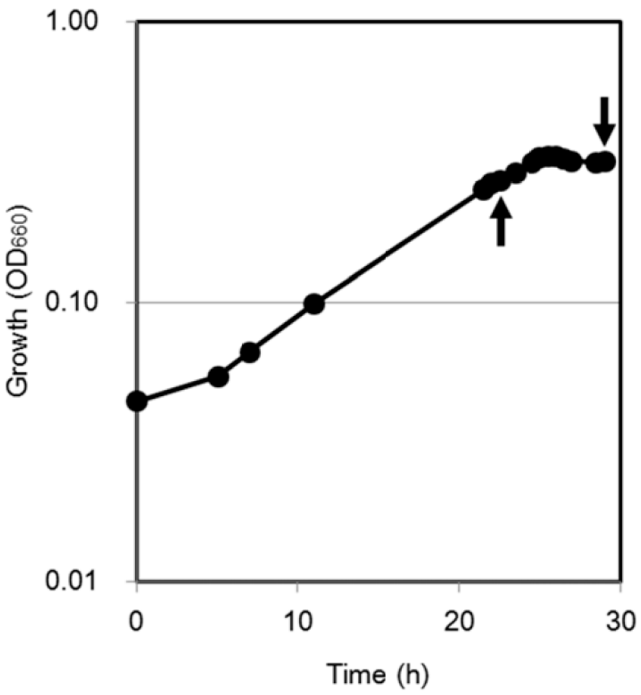

Supplement: Supplementary File 1 [file microorganisms-06-00004-s001.pdf]
